# Intervention of Astragalus Membranaceus Extract in rats of spinal cord injury: a systematic review and meta-analysis

**DOI:** 10.3389/fneur.2025.1637608

**Published:** 2025-08-01

**Authors:** Hongli Wu, Lanlan Yu, Hao Yang, Jiahao Li, Jing Deng, Zichao Zhou, Yufeng Tao, Fengjiao Chen, Leyi Zhang, Chi Zhang

**Affiliations:** Chengdu University of Traditional Chinese Medicine, Chengdu, China

**Keywords:** phytomedicine, spinal cord injury, animal experiments, meta-analysis, Huang qi

## Abstract

**Objective:**

Spinal cord injury (SCI) causes motor, sensory and autonomic dysfunction below the level of injury and its incidence is increasing every year. Astragalus Membranaceus Extract (AME) has received attention in spinal cord injury in recent years, but its specific effects in spinal cord injury are unclear.

**Methods:**

Databases of PubMed, Embase, WOS, Cochrane Library, FMRS, Clinical trial, CNKI, VIP, and WangFang were searched from their establishment to December 1, 2024 using the following terms: “Astragalus propinquus,” “Huang qi,” “*Astragalus mongholicus* Bunge,” “Spinal cord injuries,” “spinal cord diseases,” “spinal cord trauma.” To ensure comprehensiveness, the search strategy included both traditional names (Astragalus) and scientific names (Astragalus membranaceus). Only studies published in Chinese or English were included. Cross-sectional studies, survey designs, quality improvement studies, and other study designs that did not meet the inclusion criteria were excluded.

**Results:**

After screening, a total of 16 studies with 996 animals were included in the review. Astragalus Membranaceus Extract (AME) administration was associated with more significant functional recovery (mean difference [MD] = 3.68, 95% CI = 2.74, 4.62). Subgroup analyses showed the best functional recovery of the spinal cord when the dose exceeded 20 units and the duration of treatment was less than 14 days.

**Conclusion:**

Our study suggests that AME has therapeutic potential for spinal cord injured rats. Further studies are needed to determine if this can be developed into a new alternative therapy through experimental and clinical studies with larger samples.

**Systematic review registration:**

Identifier: CRD42024623721, website: https://www.crd.york.ac.uk/PROSPERO/

## Introduction

Spinal cord injury (SCI) is defined as a structural and functional impairment of the spinal cord due to a variety of etiologies, resulting in motor, sensory, and autonomic dysfunction below the plane of injury (1). The consequences of SCI are frequently protracted, encompassing a loss of function, persistent psychological stress, and a significant financial burden. The incidence of SCI has exhibited an upward trend over the past three decades (2).

In 2019, the incidence of spinal cord injury (SCI) ranged from 10.4 to 83 cases per million people annually. In the United States, the prevalence of SCI affects over 280,000 individuals, with an annual incidence of 17,000 new cases (3). Latin America reports 79,412 new cases annually (4). The lifetime cost per SCI patient is estimated to exceed $3 million, and the broader societal costs associated with SCI are challenging to quantify (5). In Canada, the annual economic burden is estimated at $2.67 billion, while the U. S. spends approximately $4 billion annually on SCI medical management (1, 6, 7).

Often secondary to motor vehicle accidents (MVAs), falls, violence, and sports injuries, SCI is more prevalent in males, with studies suggesting that its incidence peaks in younger age groups (4, 7–10). Simultaneously, the incidence of acute spinal cord injury from falls in older people is increasing (10). The impact of SCI can be expected to increase in the future as the global aging process intensifies. In the past, SCI was considered an untreatable disease (11), and even more than 20 years ago the prognosis of SCI was considered quite pessimistic (12). Even now, there has been significant progress in the development of therapeutic strategies aimed at overcoming neurodegenerative processes and minimizing secondary neuronal damage including, but not limited to, early surgical intervention, the use of corticosteroids, neurotrophic drugs, cell transplantation, and rehabilitation (10–14). Surgical intervention is essential, and early and rapid surgical intervention may reduce neural tissue damage and improve SCI outcomes (15, 16). Nevertheless, there is a modicum of controversy surrounding the selection of surgical procedures and the timing of surgery (16). The concomitant symptoms of SCI, The presence of concomitant SCI symptoms, such as pain, ulcers, sensory-motor function impairments, dysfunction of the autonomic nervous system, and neuropathic pain, among others, has been well-documented and is associated with diminished quality of life for patients (17). Methylprednisolone is widely used in the early stages of SCI due to its capacity to reduce inflammation and improve blood flow to the spinal cord. However, it can cause infections in the urinary tract, respiratory system, and wounds (15, 18). In order to address these limitations and expand treatment strategies, researchers have also explored alternative pharmacological options. 4-Aminopyridine, one of the most studied drugs, has been shown to improve central motor conduction and show improvement in neurologic signs, but the evidence supporting the effectiveness of 4-aminopyridine in reducing spasticity states after SCI is currently weak. Erythropoietin (EPO) as a potential treatment could reduce damage secondary to spinal cord injury by improving motor function, modulating antioxidant and neurogenesis, and minimizing apoptosis and inflammation, but further experimental validation is needed. In addition to this, tetrahexose monosialate ganglioside sodium salt (GM-1), riluzole, granulocyte colony-stimulating factor (G-CSF), and hepatocyte growth factor (HGF) have shown some effect in the treatment of SCI, but there are few studies on this and further observation is needed. It is worth noting that although many compounds have been tested and achieved promising results, no compound has completed phase III trials and been approved for drug administration in SCI (19–24). Cell transplantation has emerged as a particularly promising therapeutic approach in both preclinical and clinical studies. Mesenchymal stem cells, embryonic/neuronal stem cells mesenchymal stem cells (MSCs), embryonic/neuronal stem cells (ESCs), etc. have been used in immunomodulation and trophic support, repair of mechanically damaged neural tissues, and spinal cord protection. Related therapeutic strategies show great potential, but also face concerns about cell source, reproducibility of treatment, and tumor formation (25, 26). Therefore, based on the catastrophic consequences of SCI and the shortcomings of current treatment modalities, it remains important to continue to explore therapeutic ideas for SCI and to reduce the unfortunate consequences that occur with SCI.

Studies have shown that AME a leguminous plant, has various effects such as immunomodulation, antioxidant and anti-inflammatory, and has a good protective function on the nervous system. Astragalus injection can inhibit neuronal apoptosis and reduce neurotoxicity by inhibiting the activation of key factors in the death receptor pathway and mitochondrial pathway, exerting neuroprotective effects and thus alleviating cerebral ischemic injury (27, 28). A comprehensive review of extant literature reveals the identification of over 100 compounds in AME, including flavonoids, saponins, polysaccharides, and amino acids (29). Historically, Astragalus Membranaceus (AM) formulations have been utilized for millennia to promote neurological function rehabilitation following central nervous system injuries. Their efficacy has been validated in both clinical trials and animal experiments. However, due to the limitations inherent in traditional compounding methods, which lack detailed pharmacological characterization, it remains challenging to ascertain the specific role of AM or individual compounds in these processes. The aim of the current meta-analysis is to provide a systematic review and meta-analysis of the therapeutic potential of AME in the treatment of spinal cord injury as a basis for future clinical trials.

## Methods

Databases of PubMed, Embase, WOS, Cochrane Library, FMRS, Clinical trial, CNKI, VIP, and WangFang will be searched from their establishment to December 1, 2024 using the following terms: “Astragalus propinquus,” “Huang qi,” “*Astragalus mongholicus* Bunge,” “Spinal cord injuries,” “spinal cord diseases,” “spinal cord trauma.” The language was restricted to both Chinese and English. The protocol has been duly registered in the International Prospective Register of Systematic Reviews (PROSPERO, registration number: CRD42024623721). The study’s methodology aligns with the PRISMA statement for reporting systematic reviews and meta-analyses of studies (30).

### Search strategy

A comprehensive search was conducted in various databases, regardless of language, from the database’s inception to December 1, 2024, including PubMed, Embase, Web of Science, Cochrane Library, Clinical Trials, Foreign Medical Literature Retrieval Service (FMRS), China National Knowledge Infrastructure (CNKI), Wanfang Data Knowledge Service Platform, and VIP Chinese Science and Technology Journal Database. The search string included “astragalus” or “astragalus extract,” “rat (rats)” or “mouse (mice),” “spinal cord injury” or “spinal cord trauma.” A detailed description of the search strategy is provided in Additional file 1. Also, the reference lists of the selected articles were manually reviewed in order to obtain other relevant studies, and the Chinese Clinical Trial Registry (ChiCTR) was manually searched based on the same search terms. The China Clinical Trial Registry (ChiCTR; https://www.chictr.org.cn) is a Level 1 registry accredited by the World Health Organization ICTRP.

### Inclusion criteria

The inclusion criteria for the study are presented as follows: (1) The study is original full text of animal experiments with at least one separate control group; (2) The study population is animal models of SCI and the intervention drug is AME, with the specified dose; (3) The control group is animal models of SCI that are given the same dose of blank therapeutic fluid; (4) The outcomes include scores of BBB test (Basso, Beattie, and Bresnahan score), scores of inclined plate test, level of SOD (Superoxide Dismutase) and MDA, expression of Tumor necrosis factor-alpha (TNF-α), Interleukin 6 (IL-6), and interleukin-1beta (IL-1β). The original article should report one or more of the above outcomes.

### Exclusion criteria

The exclusion criteria for the study are presented as follows: (1) Reviews, clinical trials, case reports, editorials, or conference abstracts; (2) Non-original and incomplete research articles; (3) Studies using *in vitro* or ex vivo models; (4) treatment group using other therapies or combination with other interventions (such as herbal compounds, etc.); (5) The existence of concomitant interventions in the control group. Further, full texts are assessed by authors, and publications without relevant outcomes are excluded.

### Data selection

Two authors independently carried out the study selection process (L. L. Yu, J. Deng), which consisted of three main steps: removal of duplicate studies, preliminary screening based on titles and abstracts, and a more detailed screening based on full-text articles. Any disagreements regarding the final inclusion of studies were resolved through discussion with the involvement of a third author (H. Yang).

### Data extraction

Based on the inclusion and exclusion criteria, two reviewers (Z. C. Zhou, Y. F. Tao) independently extracted the following data from the confirmed studies: the first author’s name, publication year, animal model characteristics (including weight, age, modeling method of SCI, and sample size); interventions and comparisons, the course of treatment, and outcomes. If the original full text did not provide the specific values, we used the GetData Graph Digitizer v2.26 software^1^ to extract the specific data from graphs. If the included literature could not be accessed, we contacted the authors of the literature to obtain the relevant data, and if we failed to contact the authors, the relevant article was excluded. If there were any disagreements, the authors would discuss and resolve them themselves and examine them by a third investigator (H. Yang).

### Risk of bias assessment

The risk-of-bias (ROB) of each included study was assessed by two independent reviewers (J. H. Li, F. J. Chen) using the Systematic Review Center for Laboratory Animal Experimentation (SYRCLE) ROB tool (31). The tool provides 10 items involved in six aspects of bias (selection, performance, detection, attrition, reporting, and others). Studies meeting these criteria were considered low risk, while those not meeting them were deemed high risk. Studies with unclear bias descriptions were categorized as unclear risk. Any different opinions should be resolved through mutual discussions and examined by a third investigator (L. Y. Zhang).

### Statistical analysis

The means and standard deviations of the continuous variables were recorded using Microsoft Excel. The data were subsequently analyzed and visualized using Review Manager 5.3.0 and Stata 18.0 software. The heterogeneity index (I^2^) was employed to assess the extent of heterogeneity between studies. In instances where heterogeneity did not exceed 50%, a fixed-effects model was employed. Conversely, a random-effects model was utilized. Sources of heterogeneity were investigated according to varying extracts, intervention times, and intervention doses. Subgroup and sensitivity analyses were employed to identify areas of variance, and funnel plots were utilized to assess publication bias when ≥10 studies were included in the meta-analysis. A *p*-value of <0.05 was considered statistically significant. The robustness of the outcomes data was evaluated through a sensitivity analysis using the leave-one-out approach.

## Results

### Retrieve results

**Figure 1:**
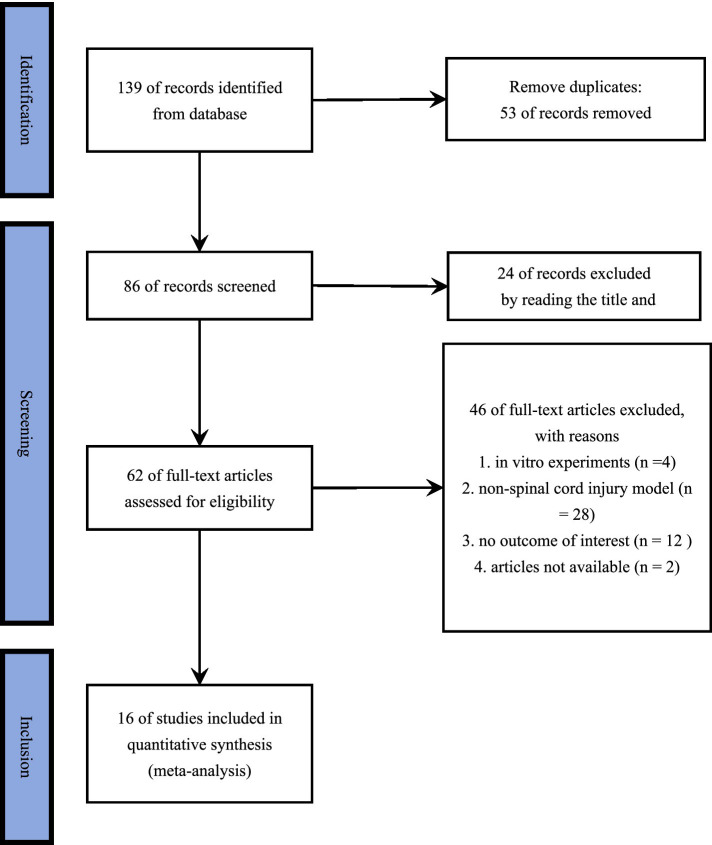
PRISMA 2020 flow diagram of study selection process. A comprehensive search was conducted using key terms across multiple databases, initially identifying 139 studies. First, 59 duplicate records were removed. Second, 24 studies were excluded based on title and abstract screening for not meeting the inclusion criteria. Third, 4 *in vitro* studies and 28 studies involving non–spinal cord injury models were excluded for the same reason. Additionally, 12 studies were removed due to missing essential data in the results, and 2 studies were excluded due to inaccessibility. Ultimately, 16 studies were included in the final analysis.

In total, 139 relevant studies were retrieved through the search. Full text review of 64 articles was performed by removing duplicated articles and reading the titles and abstracts. Of these, 48 articles were removed because they did not meet the inclusion criteria, and finally 16 randomized controlled trials were included in this meta-analysis. The flowchart of the study selection process and screening results is shown in Figure 1.

### Characteristics of the study

This meta-analysis included 16 studies published between 2006 and December 1, 2024 that utilized AME to intervene in SCI rat models. Fourteen of the studies employed Allen’s impactor approach to establish the rat model of SCI, one study utilized the PSI IH-0400 spinal cord impactor, and one study employed the mouse spinal cord compression device. All three devices are used in spinal cord crush injury modeling, which is the process of causing damage to the spinal cord through impact, compression, or stretching to simulate the effects of an external injury on the spinal cord. Specifically, the animal’s spine is physically impacted by an impact rod or impact head, etc., which creates a compression injury on the spinal cord. Nine studies used Astragalus injection (AI) as an interventional drug. In two studies, ‘Astragalus Glycoprotein’ was utilized as an intervening drug, and ‘Astragalus Polysaccharides’ was used as an interventional drug in two other studies. ‘Astragaloside IV’, ‘Total flavonoids of Astragalus’, and ‘Complanatuside A’ each had one article in the included studies. In 15 studies, the route of administration was injection. Of these, 12 studies employed intraperitoneal injection, while one study utilized caudal vein injection. One study employed the gavage method. The duration of drug intervention ranged from 1 day to 4 weeks. Table 1 provides a synopsis of the salient characteristics of the included studies.

**Table 1:** Synopsis of the salient characteristics of the included studies.

References	Animal Types	Age (weeks), weight (g)	Modeling Methods	Intervention drugs	Sample size		Intervention		Methods of administration	Duration of administration	References
					Treatment	Control	Treatment	Control			
Feng et al. (2022) (56)	Sprague Dawley rat	NR, 180–200 g	Allen’s impactor	Astragalus injection	8		8		4 mL/kg/d	Equal normal saline	Intraperitoneal injection
Liu et al. (2023) (57)	Sprague Dawley rat	NR, 230–280 g	Mouse spinal cord compression device	Astragaloside IV	10		10		40 mg/kg/d	NR	Injection
Luo (2013) (58)	Sprague Dawley rat	NR, 290–360 g	Allen’s impactor	Astragalus injection	36		36		8 g/kg/d	Equal normal saline	Intraperitoneal injection
Luo et al. (2016) (59)	Sprague Dawley rat	NR, 290–360 g	Allen’s impactor	Astragalus injection	36		36		8 g/kg/d	Equal normal saline	Intraperitoneal injection
Ren et al. (2006) (60)	Sprague Dawley rat	NR, 300–350 g	Allen’s impactor	Astragalus injection	20		20		Postoperative: 4 mL/q8h, After the first day of operation: 2 mL/kg/d	Adequate amount of normal saline	Intraperitoneal injection
Ren et al. (2009) (61)	Sprague Dawley rat	NR, 238.79 ± 20.98 g	Allen’s impactor	Astragalus injection	13		12		2 mL/kg/d	Sterile water for injection: 0.5 mL/kg/d	Intraperitoneal injection
Shi and Gao (2021) (62)	Sprague Dawley rat	6–9 W, 220–250 g	Allen’s impactor	Astragalus glycoprotein	10		10		1.0 mg/kg/d	0.9% sodium chloride injection: 1.0 mg/kg/d	Intraperitoneal injection
Shi (2021) (63)	Sprague Dawley rat	8w, 220 g	Allen’s impactor	Total flavonoids of Astragalus	8		8		50.0 mg/kg/d	Equal normal saline	Intraperitoneal injection
Wang et al. (2020) (64)	Sprague Dawley rat	NR, 200–225 g	Allen’s impactor	Astragalus glycoprotein	20		20		1 mg/kg/d	Equal 0.9% sodium chloride injection	Intraperitoneal injection
Xu et al. (2024) (65)	C57BL/6 J mice	6–8w, 18–20 g	PSI IH-0400 spinal cord impactor	Complanatuside A	6		6		50 mg/kg/d	Equal normal saline	Gavage
Yang et al. (2010) (66)	Sprague Dawley rat	NR, 220 ± 20 g	Allen’s impactor	Astragalus injection	12		12		12 h postoperative: 1 mL, 36 h postoperative: 1 mL, 5 to 9 days postoperative: 1 mL	Equal normal saline	Caudal vein injection
Yu et al. (2010) (67)	Wistar rat	4w, 80 ± 10 g	Allen’s impactor	Astragalus injection	22		22		1.2 mL/100 g/d	NR	Intraperitoneal injection
Zhang and Lv (2011) (68)	Sprague Dawley rat	NR, 300 ± 50 g	Allen’s impactor	Astragalus injection	48		48		30 min postoperative: 4 mL, 1 h postoperative: 2 mL AI + 2 mL NS, 4 h postoperative: 2 mL AI + 2 mL NS,	Equal amounts of saline given at the same time	Intraperitoneal injection
Zhang and Tang (2014) (69)	Sprague Dawley rat	NR, 220–250 g	Allen’s impactor	Astragalus injection	10		10		2 mL/kg/d	Normal saline: 0.5 mL/kg/d	Injection
Zheng et al. (2016) (70)	Sprague Dawley rat	NR, 200–220 g	Allen’s impactor	Astragalus polysaccharides	30		30		30 min postoperative: 4 mL, From the first day after surgery: 10 mL/kg/d	NR	Intraperitoneal injection
Zheng (2017) (71)	Sprague Dawley rat	NR, 200–220 g	Allen’s impactor	Astragalus polysaccharides	30		30		30 min postoperative: 4 mL, From the first day after surgery: 10 mL/kg/d	Equal amounts of saline given at the same time	Intraperitoneal injection

NR, not reported; g, weight unit (grams) or dose per kilogram depending on context; Allen’s impactor, standardized weight-drop spinal cord injury model; mouse spinal cord compression device, mechanical compression injury model; Astragalus injection, aqueous extract of Astragalus membranaceus; Astragalus polysaccharides/flavonoids/glycoprotein, purified bioactive compounds from Astragalus; IP injection, intraperitoneal injection; NS, normal saline (0.9% sodium chloride solution); mL/kg/d, volume administered per kilogram per day; mg/kg/d, mass administered per kilogram per day; complex dosing (e.g., Ren, 2006), indicates dose variation at different postoperative time points; sample size, total number of animals per group (treatment + control).

### Study quality

**Figure 2:**
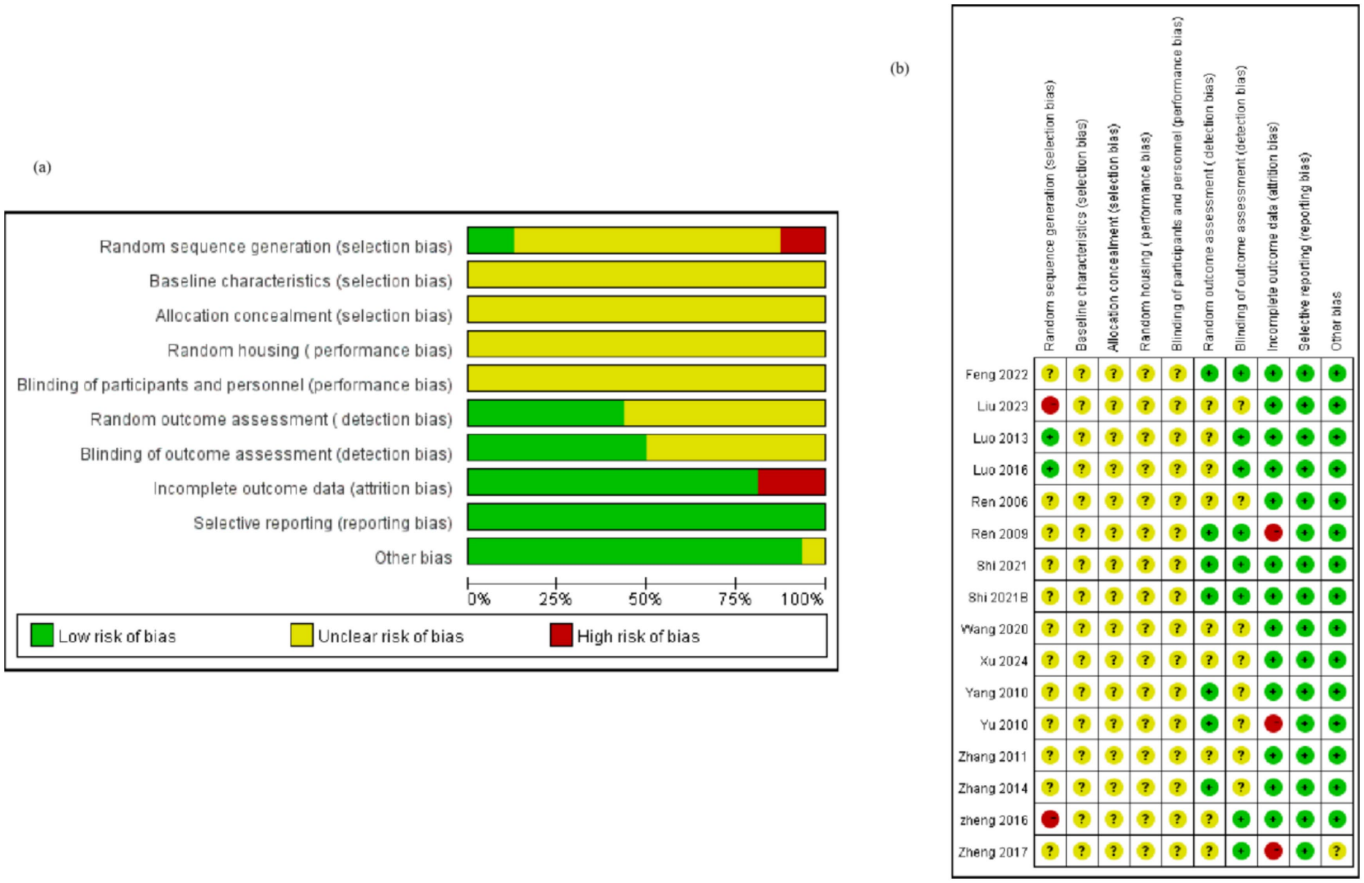
Quality of the included studies. Selection bias includes random sequence generation, baseline characteristics, and allocation concealment; performance bias includes random housing and blinding of participants and personnel; detection bias includes random outcome assessment and blinding of outcome assessment; attrition bias: incomplete outcome data; reporting bias: selective reporting; Other biases: new animals added to the experimental group, etc. Bias analysis indicates that most of the included studies had deficiencies in baseline characteristics, allocation concealment, and randomization. Related studies need to be improved in terms of the randomization process and data integrity control. However, in terms of reporting completeness and control of other potential biases, the included studies met the relevant standards.

Two studies utilized the random number table method for random assignment of rats (12.5%), three studies did not specify whether animals were randomly assigned (18.75%), and 11 studies merely mentioned “randomized” without providing detailed information (68.75%). Notably, none of the studies referenced the following protocols: baseline characteristics, allocation concealment, or blinding of trial caregivers. While three studies mentioned the housing environment of the rats (18.75%), they did not adhere to the Random Housing protocol. Five studies (31.25%) mentioned random selection of rats for testing, and nine studies (56.25%) employed third-party testing or double-blinding to avoid subjective scoring bias. Concerning incomplete outcome data, one study reported animal deaths after modeling (6.25%), and another study substituted new animals for those that failed to model (6.25%), which may have introduced additional sources of bias (Figure 2).

### Meta-analysis meta

#### Scores of BBB test

**Figure 3:**
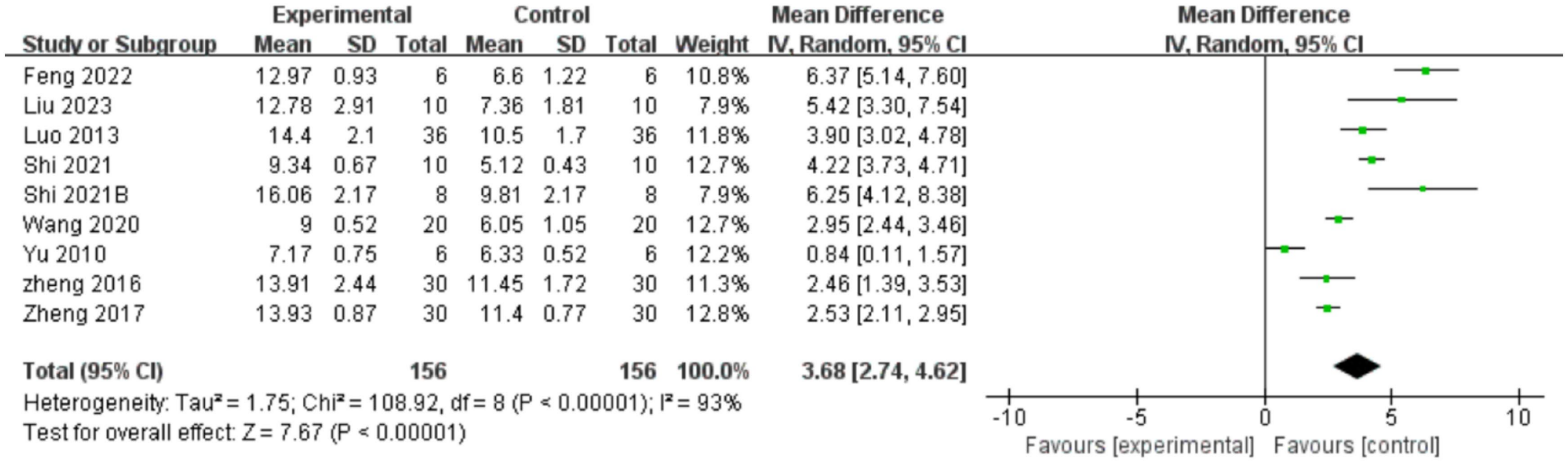
Forest plot of BBB test scores for the AME group vs. the control group. The BBB test included a total of 9 studies, with 156 rats in the AME group and 156 in the control group, totaling 312 rats. Mean Difference (MD): 3.68, 95% confidence interval (95% CI): [2.74, 4.62], and Test for overall effect: *Z* = 7.67, (*p* < 0.00001), indicating a significant statistical difference between the experimental and control groups, with the AME showing superior efficacy. The heterogeneity test showed Tau^2^ = 1.75, Chi^2^ = 108.92, df = 8, (*p* < 0.00001), and *I*^2^ = 93%, indicating high heterogeneity among the included studies.

A total of nine studies indicated that AME enhanced BBB test scores (Figure 3), with the results demonstrating that the scores of BBB test in the AME treatment group exhibited a significant increase compared to the control group (MD = 3.68, 95% CI = 2.74, 4.62). Due to the considerable heterogeneity observed (*I*^2^ = 93%), a more thorough analysis was conducted, examining subgroups based on the type of extract, drug unit, drug dose, and time of intervention. The results demonstrated that scores of the BBB test were elevated in the rat models of SCI treated with AME in comparison to the control group. The maximum scores were observed with AI doses greater than 20 units and treatment durations not exceeding 14 days. No subgroup could reduce the heterogeneity (Table 2).

**Table 2:** Subgroups of BBB test scores for the AME group vs. the control group.

Subgroup	Eligible Studies	Intervention group (n)	Control group (n)	Standardized Mean Difference [95% CI]	*p*-value	Heterogeneity test	Total heterogeneity test
Treatment time							
≤14 days	5	66	66	4.47 [2.09, 6.85]	0.0002	*P* < 0.00001, *I*^2^ = 95%	*p* < 0.00001, *I*^2^ = 93%
>14 days	4	90	90	3.07 [2.21, 3.93]	<0.00001	*P* < 0.00001, *I*^2^ = 93%	
Intervention drugs							
Astragalus injection	3	48	48	3.67 [0.61, 6.74]	0.02	*P* < 0.00001, *I*^2^ = 97%	*P* < 0.00001, *I*^2^ = 94%
Astragalus glycoprotein	2	30	30	3.59 [2.34, 4.83]	<0.00001	*p =* 0.0005, *I*^2^ = 92%	
Astragalus polysaccharides	2	60	60	2.52 [2.13, 2.91]	<0.00001	*p =* 0.90, *I*^2^ = 0%	
Drug dose							
<10 units	3	36	36	4.40 [2.94, 5.86]	<0.00001	*P* < 0.00001, *I*^2^ = 93%	*p* < 0.00001, *I*^2^ = 93%
10–20 units	3	66	66	1.94 [0.79, 3.09]	<0.00001	*p =* 0.0004, *I*^2^ = 87%	
>20 units	3	54	54	4.93 [3.40, 6.46]	<0.00001	*p =* 0.08, *I*^2^ = 60%	
Unit of dose							
Quality Unit (mg/kg)	4	74	74	4.00 [3.06, 4.93]	<0.00001	*P =* 0.0004, *I*^2^ = 83%	*P* < 0.00001, *I*^2^ = 93%
Volume Unit (mL/kg)	5	82	82	3.38 [3.40, 6.46]	<0.00001	*P* < 0.00001, *I*^2^ = 94%	

SMD, standardized mean difference; CI, confidence interval; IP, intraperitoneal injection; NS, normal saline (0.9% sodium chloride solution); g, absolute weight (grams) or dose per kilogram depending on context; mL/kg/d, volume administered per kilogram per day; mg/kg/d, mass administered per kilogram per day; Allen’s impactor, standardized spinal cord injury model using weight-drop mechanism; mouse spinal cord compression device, mechanical compression model; Astragalus injection, aqueous extract of Astragalus membranaceus; Astragalus polysaccharides/flavonoids/glycoprotein, purified bioactive components from Astragalus; NR, not reported; sample size, total number of animals per group (treatment + control); unit, operational definition unclear (e.g., 1 unit = X mg extract); I^2^, heterogeneity index (moderate: 50–75%; high: ≥75%); random-effects model, applied when heterogeneity is substantial; sensitivity analysis, recommended for high-heterogeneity subgroups; publication bias, not assessed (e.g., funnel plot, Egger’s test); efficacy hierarchy, high dose > short duration > mg/kg > injection; robustness, highest in Astragalus polysaccharide subgroup (*I*^2^ = 0%).

BBB test: used to assess recovery of motor function in small animals after spinal cord injury, usually assessed at 1, 2, and 4 weeks after spinal cord injury. Animals are scored based on indicators of gait, motor coordination, and improvement in posture and gait, with scores ranging from 0 to 21, with 0 indicating complete paralysis and 21 indicating complete recovery (55).

#### Scores of inclined plate test

**Figure 4:**
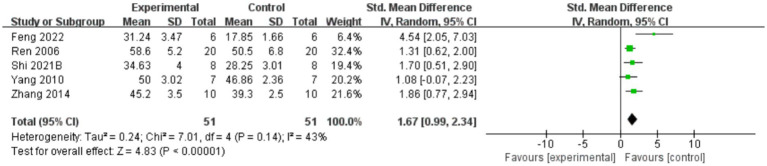
Forest plot of inclined plate test scores for the AME group vs. the control group. A total of 6 studies were included, with 51 cases in the AME group and 51 cases in the control group, for a total sample size of 102 cases. The standardized mean difference (SMD) was calculated as 1.67, 95% CI: [0.99, 2.34], and Test for overall effect: *Z* = 4.83, (*p* < 0.00001), indicating a significant statistical difference between the experimental and control groups, with the AME group showing superior efficacy. Tau^2^ = 0.24, Chi^2^ = 7.01, df = 4 (*p* = 0.14), and *I*^2^ = 43%, indicating moderate heterogeneity among the studies.

A total of five reports indicated that AME enhanced inclined plate test scores (Figure 4), with the results demonstrating that AME exhibited a more pronounced effect in the treatment group compared to the control group (SMD = 1.67, 95% CI = 0.99, 2.34).

The inclined plate test is used to assess muscle strength and locomotion in mice or rats, especially upper and lower limb support. Assessments are generally performed days to weeks after a spinal cord injury, depending on the study design, with common assessment time points being 1, 2 and 4 weeks post-injury. In the experiment, the animal is placed on an inclined plane and the slope is gradually increased until the animal is unable to maintain balance or slides downward. The test assessed the animal’s locomotor ability by recording the maximum angle it could withstand. Lower slopes (20°) usually indicate a more severe spinal cord injury or poorer muscle recovery. Higher slopes indicate less severe injuries or better recovery.

#### Inspection results

**Figure 5:**
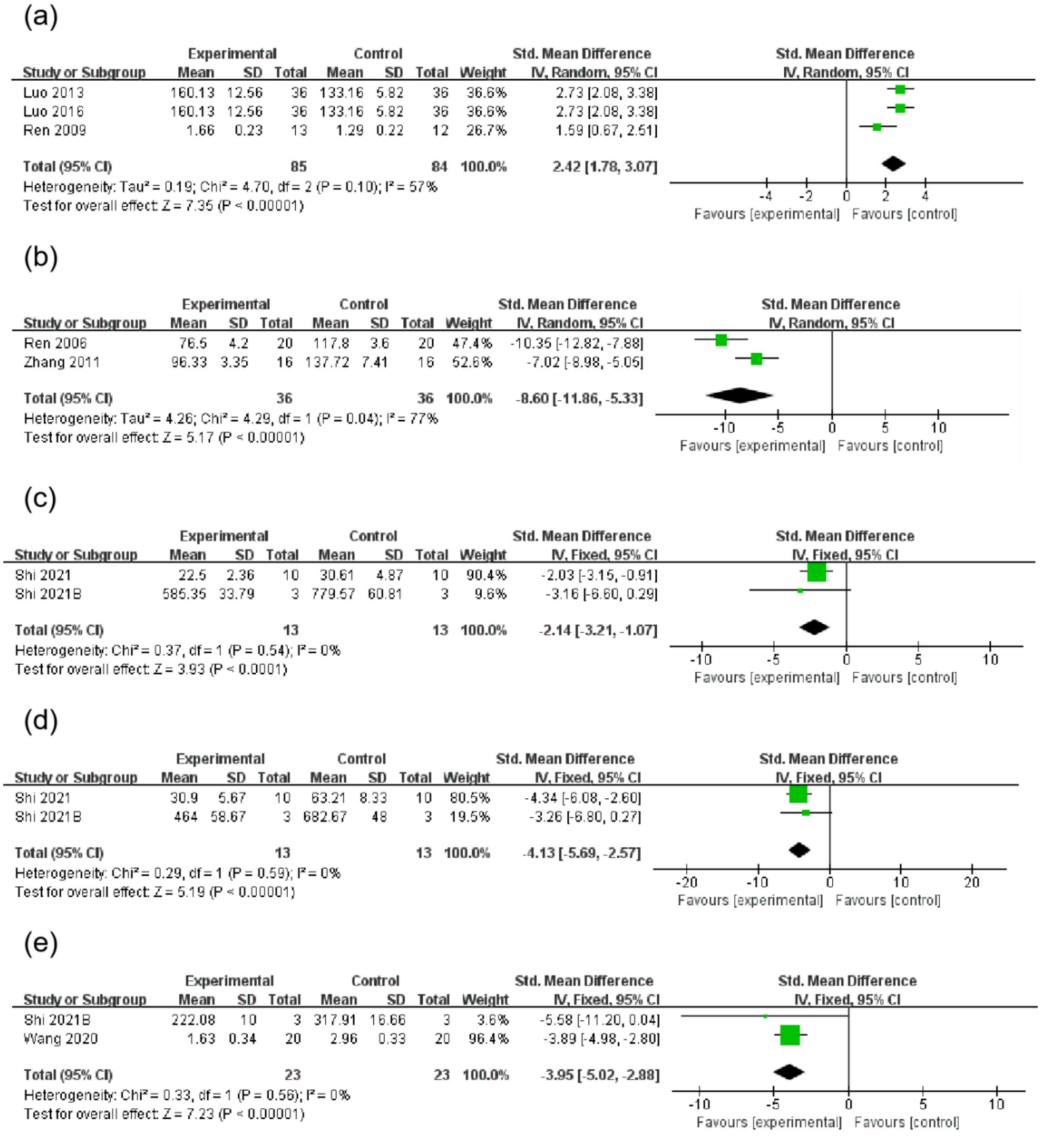
Forest plot of inspection results for the AME group vs. the control group. **(a)**: SOD, **(b)**: MDA, **(c)**: IL-6, **(d)**: TNF-α, **(e)**: IL-1β. **(a)** Three studies were included, with 85 rats in the AME group and 84 rats in the control group, totaling 169 rats. SMD = 2.42, 95% CI: [1.78, 3.07], *Z* = 7.35 (*p* < 0.00001), indicating that the AME group had a superior effect. Heterogeneity: Tau^2^ = 0.19, Chi^2^ = 4.70, df = 2 (*p* = 0.10), *I*^2^ = 57%, indicating moderate heterogeneity. **(b)** Two studies were included, with 36 rats in the AME group and 36 rats in the control group, totaling 72 rats. SMD = −8.60, 95% CI: [−11.86, −5.33], *Z* = 5.17 (*p* < 0.00001), indicating that the AME group had a superior effect. Heterogeneity: Tau^2^ = 4.26, Chi^2^ = 4.29, df = 1 (*p* = 0.04), *I*^2^ = 77%, indicating high heterogeneity. **(c)** Two studies were included, with 13 rats in the AME group and 13 rats in the control group, totaling 26 rats. SMD = −2.14, 95% CI: [−3.21, −1.07], *Z* = 3.93 (*p* < 0.0001), indicating that the AME group had a superior effect. Heterogeneity: Chi^2^ = 0.37, df = 1 (*p* = 0.54), *I*^2^ = 0%, indicating no heterogeneity. **(d)** Two studies were included, with 13 rats in the AME group and 13 rats in the control group, totaling 26 rats. SMD = −4.13, 95% CI: [−5.69, −2.57], *Z* = 5.19 (*p* < 0.00001), indicating that the AME group had a superior effect. Heterogeneity: Chi^2^ = 0.29, df = 1 (*p* = 0.59), *I*^2^ = 0%, indicating no heterogeneity. **(e)** Two studies were included, with 23 rats in the AME group and 23 rats in the control group. SMD = −3.95, 95% CI: [−5.02, −2.88], *Z* = 7.23 (*p* < 0.00001), indicating that the AME group had a superior effect. Heterogeneity: Chi^2^ = 0.33, df = 1 (*p* = 0.56), *I*^2^ = 0%, no heterogeneity.

A total of three studies reported that AME increased SOD levels (SMD = 2.42, 95%CI = 1.78, 3.07). Two studies reported that AME decreased MDA levels (SMD = −8.60, 95%CI = −11.86, −5.33). Two studies reported that AME decreased IL-6 levels (SMD = −2.14, 95%CI = −3.21, −1.07), two studies reported that AME reduced TNF-α levels (SMD = −4.13, 95% CI = −5.69, −2.57) and two studies reported that AME reduced IL-1β levels (SMD = −3.95, 95% CI = −5.02, −2.88) (Figure 5 and Table 3).

**Table 3:** Details on relevant literature.

Study title	Author	Year	Tissue analyzed	Was the sample taken prior to euthanasia or after	Route through which these results were obtained	Survival time post-SCI
Effects of astragaloside IV on spinal cord injury and apoptosis of spinal cord glial cells	Ming Liu	2023	Spinal cord tissue	Euthanasia	Spinal cord oxidative stress marker kit	14 days
Laboratory study and clinical observation of Astragalus root on influence of nerve function after spinal cord injury	Junpu Luo	2013	Blood serum	open the chest and draw blood	Superoxide dismutase (SOD) activity assay	1, 3, 7 and 28 days
Effects and significance of Astragalus on SOD activity and GFAP expression in rats with spinal cord injury	Junpu Luo	2016	Blood serum	Open the chest and draw blood	Superoxide dismutase (SOD) activity assay	1, 3, 7 and 28 days
Neuro-protective effect of Astragalus root on experimental injury of spinal cord in rats	Xiansheng Ren	2006	Spinal cord tissue	Euthanasia	Xanthine oxidation and thiobarbituric acid chemiluminescence	7 days
Experimental Study of Huangqi in Rat Acute Spinal Cord Injury	Rui Zhang	2011	Spinal cord tissue	Euthanasia	Xanthine oxidation and thiobarbituric acid chemiluminescence	1, 4 and 8 h
Experiment study about astragale injection treatment on acute spinal cord injury	Zhihong Ren	2009	Spinal cord tissue	Euthanasia	Spinal cord oxidative stress marker kit	21 days

SCI, spinal cord injury; euthanasia, humane endpoint conducted prior to sampling in compliance with ethical guidelines; open the chest and draw blood, thoracotomy and cardiac puncture performed after deep anesthesia or euthanasia; SOD activity assay, superoxide dismutase activity assay to assess antioxidant capacity; oxidative stress marker kit, quantitative detection of oxidative stress indicators such as MDA and GSH; xanthine oxidation + TBA chemiluminescence, lipid peroxidation detection via xanthine oxidase system and thiobarbituric acid method; survival time, time interval from SCI modeling to final sampling; spinal cord tissue, injured-site spinal cord tissue samples; blood serum, centrifuged serum obtained from collected blood.

#### Sensitivity analysis and publication bias

**Figure 6:**
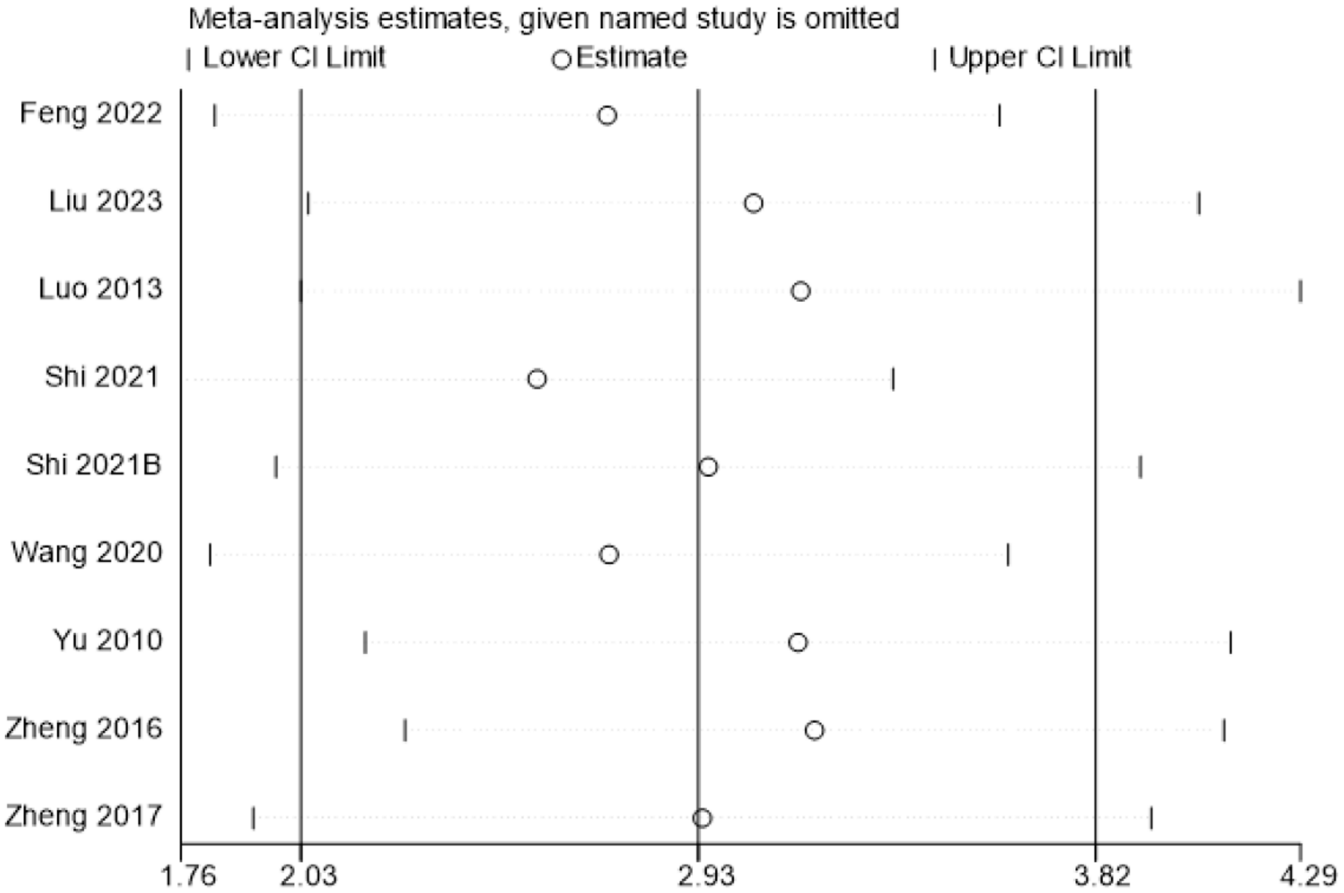
Sensitivity analysis of BBB test scores. CI, confidence interval. Estimate: after excluding various studies, the effect sizes exhibited positional changes. However, the overall did not show extreme deviation. Lower and Upper CI Limit: following the exclusion of studies, there were no dramatic changes in the upper and lower limits. As depicted in figure, the effect sizes and confidence intervals did not undergo substantial changes following the exclusion of a single study. This suggests that the results were relatively stable.

Excluding any study with no significant changes in the heterogeneity index and 95% confidence intervals indicated non-significant differences between studies. This result demonstrates that the results of meta-analysis were stable and the robustness of the meta-analysis results was affirmed. According to the guidelines of the Cochrane Collaboration’s guidance, sensitivity analyses, funnel plots, and Egger’s tests were not performed for the other outcome indicators due to the small number of included studies (Figure 6).

## Discussion

The high prevalence of SCI carries a significant burden, often accompanied by permanent impairment of motor and autonomic nervous system function, severely affecting the quality of life of patients and their families. The etiology of SCI encompasses a wide spectrum of etiologies, including contusion, compression, transection, and ischemia–reperfusion injury, among others. To achieve a more authentic simulation of SCI, the use of animal models, including rats, cats, dogs, rabbits, and orangutans, has been employed. Rats have been the preferred model due to their cost-effectiveness, vascular anatomy, and similarity to human physiology (32). The BBB test is a reliable and standardized method for evaluating the effectiveness of treatments in rats with SCI (33). This meta-analysis examined the efficacy of AME in treating a rat model of SCI, and the results demonstrated that AME was more effective in motor function recovery. A subsequent subgroup analysis revealed that the optimal recovery of motor function was attained when the AI dose exceeded 20 units and the treatment duration was less than 14 days. Furthermore, the analysis revealed that AME significantly promoted SOD expression, while concurrently reducing MDA levels and inhibiting IL-6, TNF-α, and IL-1β expression. This finding suggests that the therapeutic potential of AME in improving these parameters may be valuable in SCI rats.

Surgical intervention constitutes a pivotal component of treatment for SCI. The fundamental objective of surgical intervention is to alleviate compression on the spinal cord, thus restoring stability and creating a conducive anatomical environment for the restoration of function. The primary objective of early surgery is to rapidly alleviate spinal cord compression, effectively mitigate mechanical damage to the spinal cord, enhance local blood circulation, minimize the risk of secondary damage to neurons and glial cells, and forestall further deterioration of the injury. Conversely, delayed surgery emphasizes the reconstruction and consolidation of spinal stability, thereby establishing a stable environment conducive to spinal cord recovery (10, 34, 35).

Secondary injury occupies a significant position in the progression of SCI, leading to lesions in otherwise uninjured tissues adjacent to the initial site of injury. Related studies have identified oxidative stress as a hallmark of the second stage of SCI injury (1, 36, 37). The dynamic equilibrium between reactive oxygen species (ROS) and antioxidants during SCI recovery is crucial for sustaining normal cellular physiological function (38). Numerous studies have demonstrated that mitigating oxidative stress constitutes a viable therapeutic strategy in the context of SCI. For instance, drugs such as the glucocorticoid steroid, methylprednisolone, have demonstrated significant antioxidant activity and improved the disease process in SCI (39, 40). The present meta-study demonstrated that AME significantly increased antioxidant levels with reduced levels of oxidative stress markers, highlighting the potent antioxidant properties.

A significant pathological factor in SCI is the inflammatory response (41). The infiltration of immune cells and inflammatory cytokines promotes neuronal inflammation, which in turn impedes nerve repair through a series of responses (1). For instance, large plasma-derived molecules are more likely to traverse the cell membrane in an inflammatory state, increasing the probability of edema (1, 42, 43). Edema of the nerve tissue, in turn, elevates interstitial pressure, which compresses the surrounding vasculature and consequently leads to or exacerbates ischemia (44). Indeed, each factor is interconnected in the disease process of SCI, with oxidative stress activating inflammatory mechanisms causing secondary damage (45). Other cellular events, such as inflammation killing oligodendrocytes leading to demyelination and, in severe cases, permanent neurological deficits (13, 33, 46), also play a role. Consequently, clinicians must take the comprehensive course of the disease into account when devising a treatment strategy. This underscores the necessity of controlling inflammation and oxidative stress factors to effectively manage secondary damage following SCI (47, 48).

The apoptotic cascade initiated by TNF-*α* after SCI has been demonstrated (49), and its use as a therapeutic target to alleviate the level of inflammation after SCI has been used as one of the many therapeutic strategies for SCI (50, 51). Other relevant studies have confirmed that IL-1β not only induces neuronal apoptosis after SCI via the p38MAPK signaling pathway (52), but also induces upregulation of IL-6, enhances vascular permeability, and induces neural stem cells to differentiate into astrocytes, which can result in the formation of neuroglial cell scarring (53). Furthermore, IL-1β and IL-6 have been observed to induce inducible nitric oxide synthase (iNOS) in neurons, leading to the overproduction of nitric oxide (NO). This excess NO contributes to the onset of necrotic and apoptotic cell death, resulting in the damage of autonomic, motor, sensory, and other functional behaviors (54).

As the primary outcome indicator in this study, BBB score showed significant heterogeneity in the combined analysis. We attempted to explore potential sources of heterogeneity through subgroup and sensitivity analyses; however, conventional grouping variables such as intervention duration, extract composition, and dosage units proved insufficient to account for the observed heterogeneity. After revisiting the nadir criteria and risk of bias, we hypothesized that biological differences in experimental animals might be one of the important confounding factors.

We first conducted an exploratory exclusion analysis based on rat body weight. Heterogeneity decreased after excluding studies weighing less than 200 g (*I*^2^ = 81%); further exclusion of studies weighing less than 300 g resulted in a further decrease in heterogeneity (*I*^2^ = 70%). Although overall heterogeneity still did not fall to the desired level, this trend suggests that weight differences may be an important source of heterogeneity. Second, we examined the effect of gender. After excluding studies using female rats, no significant change in heterogeneity was observed, suggesting that gender may have less of an effect on spinal cord injury recovery in the context of this study. Subsequently, we analyzed the rats in groups according to their age. The results showed that the heterogeneity in the 8–9 weeks old group was zero (*I*^2^ = 0), while the adult (8–12 weeks) and juvenile (8 weeks) groups exhibited higher heterogeneity (*I*^2^ = 83 and 96%), respectively, further suggesting that age is one of the key factors influencing the consistency of the effect.

In addition, we attempted to analyze the source of heterogeneity in terms of modeling conditions. Although most studies used the 10.0 g × 25 mm weight drop method for standardized injury modeling, heterogeneity remained high after excluding studies with inconsistent modeling parameters (*I*^2^ = 94%). When analyzing the injured segments, it was found that although most studies focused on the T10 segment, heterogeneity did not significantly improve after excluding non-T10 studies (*I*^2^ = 92%). Due to the limited number of studies related to other segments, we were unable to conduct further valid subgroup analyses at this time.

Collectively, unavoidable biological variation (e.g., body weight, age) and experimental design differences (e.g., modeling approach, segment selection) in animal experiments constitute an important source of heterogeneity. We have fully presented the above issues in the Discussion and suggested that future studies should further strengthen the control of animal model characteristics, standardization of experimental procedures, and reporting practices. Although the limited number of studies on some secondary outcomes may affect the statistical rigor, we believe that this research remains necessary and valuable. Despite the statistical inconsistency of the pooled results, the findings still offer meaningful directional insights, especially in the current context where high-quality systematic reviews in this field remain scarce. These results may serve as a useful reference for future research and evidence synthesis.

### Strengths and limitations

To the best of our knowledge, the present meta-study is the first comprehensive evaluation of randomized controlled trials of AME for the treatment of SCI rats. Conventional databases were reviewed, and an additional review was conducted at Clinical Trials and ChiCTR to ensure the inclusion of all relevant studies. The analysis was further refined through subgroup analysis, which investigated the influence of dosage and treatment duration on recovery of function after SCI. However, as is the case with other meta-analyses, this study was similarly limited by low-quality data and deficiencies in the methodological quality of the studies. The databases searched were limited to Chinese and English, potentially missing studies written in other languages. The study’s small sample size also imposed limitations. Future studies should focus more on AME interventions in SCI modeling and should prioritize enhancing the methodological quality of the study, such as implementing a more rigorous blinding strategy.

## Conclusion

This meta-analysis demonstrated that by analyzing the BBB test scores, inclined plate test scores, and changes in biochemical indexes in the SCI rat models, AME could effectively promote the recovery of motor function, increase the levels of antioxidants with reduced levels of oxidative stress markers, and reduce the degree of inflammation after SCI, demonstrating that AME has therapeutic potential for SCI rats. In light of the extant data on the subject, it is concluded that AME is a promising phytomedicine for promoting the recovery of motor function and improving the levels of oxidative stress and inflammation in SCI. The potential for this to serve as a novel therapeutic alternative warrants further investigation.

## Data Availability

The original contributions presented in the study are included in the article/supplementary material, further inquiries can be directed to the corresponding author.

## Footnotes

^1^ getdata-graph-digitizer.com
